# Adult-onset Still’s disease complicated by Guillain-Barré syndrome

**DOI:** 10.1093/rheumatology/kead125

**Published:** 2023-03-17

**Authors:** Nicola Farina, Daniel Arroyo-Sánchez, Corrado Campochiaro, Lorenzo Dagna, Alessandro Tomelleri

**Affiliations:** Unit of Immunology, Rheumatology, Allergy and Rare Diseases, IRCCS San Raffaele Hospital, Milan, Italy; Department of Immunology, Hospital Universitario, Madrid, Spain; Instituto de Investigación Sanitaria, Hospital Universitario, Madrid, Spain; Unit of Immunology, Rheumatology, Allergy and Rare Diseases, IRCCS San Raffaele Hospital, Milan, Italy; Vita-Salute San Raffaele University, Milan, Italy; Unit of Immunology, Rheumatology, Allergy and Rare Diseases, IRCCS San Raffaele Hospital, Milan, Italy; Vita-Salute San Raffaele University, Milan, Italy; Unit of Immunology, Rheumatology, Allergy and Rare Diseases, IRCCS San Raffaele Hospital, Milan, Italy; Vita-Salute San Raffaele University, Milan, Italy

Rheumatology key message

- Guillain-Barré syndrome is a rare complication of Adult-onset Still’s disease that requires specific treatment.

Dear Editor, Adult-onset Still’s disease (AOSD) is a rare autoinflammatory disease with a heterogeneous clinical spectrum [1]. Although rare, neurological involvement, usually in the context of a concomitant macrophage activation syndrome (MAS), can also be part of such spectrum [2], with meningitis and encephalitis being the most common manifestations [3]. While Miller Fisher syndrome has been previously described in a patient with AOSD in this journal [4], classical Guillain-Barré syndrome (GBS) has never been reported in this clinical setting. Herein, we describe the case of a patient affected by AOSD who developed GBS as a complication of a disease flare. Informed consent has been obtained.

A 61-year-old gentleman presented to our rheumatology outpatient clinic with a 3-day history of paraesthesia over the perioral area and lower limbs. He also reported inability of smiling and closing both eyes. Clinical examination revealed disappearance of nasolabial folds, lagophthalmos, and lower limbs hyporeflexia.

Two years before, the patient had been admitted to the internal medicine ward due to persistent fever up to 40°C, pharyngitis and migrating arthralgias. At that time, blood tests revealed marked elevation of CRP, 206 mg/l, ferritin (14 000 ng/ml), and increased liver enzymes (AST 156 U/l, ALT 71 U/l). 18-FDG PET scans showed significant uptake of multiple lymph nodes but no other abnormalities. Subsequent tests excluded oncological, infective and autoimmune aetiologies. Hence, a diagnosis of AOSD was formulated. The patient was started on high-dose glucocorticoids (i.e. prednisone, 1 mg/kg) and clinical and biochemical remission was obtained. However, upon glucocorticoids tapering, the patient experienced a disease flare which required the addition of the interleukin-1 receptor antagonist anakinra at a dose of 100 mg daily. Anakinra treatment was highly effective and a steroid-free remission, maintained for over a year, was achieved.

One week before the current presentation, the patient was urgently reviewed at our clinic due to a 2-week history of high fever. Full blood cell count, coagulation panel and triglycerides’ level were within normal range; however, levels of CRP, ferritin, AST and ALT were increased (53 mg/l, 4000 ng/ml, 257 U/l and 290 U/l, respectively). Hepatotropic viruses’ serologies and a SARS-CoV-2 swab were negative. Abdomen CT did not reveal any abnormality, while an 18-FDG PET scan showed increased uptake of neck lymph nodes with reactive ultrasound features. A diagnosis of AOSD flare was made and prednisone 25 mg daily was re-started. Anakinra therapy was kept unchanged. Although fever promptly resolved, seven days later the aforementioned neurologic manifestations occurred. The patient was therefore admitted to the neurology ward to perform additional tests.

Head CT scan was unremarkable. An electromyography showed bilateral peripheral facial and lower limbs neuropathy consistent with demyelinating polyradiculoneuropathy. Cerebrospinal fluid analysis revealed increased levels of protein (143 mg/dl) and nucleated cells (30/µL). Because no sign of infection or other neurologic diseases were detected, a diagnosis of GBS complicating the recent AOSD flare was made. Antibodies against ganglioside GM1 tested negative, as did stool culture and serologies for recent EBV, SARS-CoV-2 and CMV infections. The patient was treated with intravenous immunoglobulin at the dosage of 1 g/kg administered over three days and neurologic manifestations promptly improved. Once discharged, prednisone was slowly tapered and anakinra was switched to the monoclonal antibody against IL-1β canakinumab. One month after, neurological manifestations had completely resolved and no signs or symptoms of active AOSD were noticed.

To our knowledge, this is the first report of classical GBS complicating the course of an AOSD patient. However, this combination is not surprising, as it is well known that both diseases share a possible viral trigger and dysregulation of the immune system [5, 6]. This pathogenic common background can explain such a peculiar association.

Indeed, it may be argued that the concurrence of these two diseases may just have been a coincidence without any causal relationship. In fact, GBS was not synchronous with the other manifestations of AOSD and had never occurred during previous flares. However, neurologic manifestations are known to possibly complicate AOSD later during the disease course, without a tight temporal connection with other symptoms [4]. Interestingly, neurologic manifestations occurred despite the ongoing prednisone treatment course, which was instead effective on systemic inflammation. Nonetheless, glucocorticoids do not represent an effective therapy for GBS [7] and, as shown by previous reports [4], intravenous immunoglobulins are always required to treat this condition. This therapeutic approach led to a very swift improvement of GBS manifestations in our patient, possibly due to the timely (i.e. within one week of symptoms’ onset) administration.

Of note, while neurologic complications of AOSD have been frequently described to develop in concomitance with MAS [3], in this case there were no signs of this life-threatening manifestation.

In conclusion, our report underlines the importance of ruling out a GBS diagnosis when a patient with AOSD presents with neurological manifestations, as a late diagnosis of this syndrome may come with important sequelae [8].

## Data Availability

Data are available upon reasonable request by any qualified researchers who engage in rigorous, independent scientific research, and will be provided following review and approval of a research proposal and Statistical Analysis Plan (SAP) and execution of a Data Sharing Agreement (DSA). All data relevant to the study are included in the article.
